# Autoimmune osteomalacia: a novel FGF23-related hypophosphatemic osteomalacia

**DOI:** 10.1007/s00774-026-01697-0

**Published:** 2026-02-14

**Authors:** Yoshitomo Hoshino, Nobuaki Ito

**Affiliations:** 1 Division of Nephrology and Endocrinology, The University of Tokyo Hospital, Tokyo, Japan; 2 Osteoporosis Center, The University of Tokyo Hospital, Tokyo, Japan; 3 Division of Therapeutic Development for Intractable Bone Diseases, Graduate School of Medicine and Faculty of Medicine, The University of Tokyo, 7-3-1 Hongo, Bunkyo-ku, Tokyo, 113-8655 Japan

**Keywords:** FGF23, Osteomalacia, PHEX, Autoantibody, Burosumab, Tumor-induced osteomalacia

## Abstract

**Background:**

Fibroblast growth factor 23 (FGF23)-related hypophosphatemic rickets/osteomalacia arises from excessive FGF23 activity, with X-linked hypophosphatemia (XLH) and tumor-induced osteomalacia (TIO) as the most common congenital and acquired forms, respectively. However, in a substantial subset of patients with acquired FGF23-related hypophosphatemic osteomalacia, phosphaturic mesenchymal tumors (PMTs) remain undetectable despite extensive imaging studies. A recent study identified autoantibodies against PHEX, the gene responsible for XLH, in 5 of 13 patients with acquired FGF23-related osteomalacia without detectable PMTs, thereby defining a novel disease entity termed autoimmune osteomalacia (AIO). Clinically, AIO presents with milder disease activity than TIO, comparable in severity to XLH.

**Findings:**

Some patients exhibited concomitant autoimmune disorders, and whole-genome sequencing revealed rare variants in autoimmune susceptibility genes, suggesting a genetic predisposition. Therapeutic options include burosumab and, potentially, immunosuppressive therapy such as glucocorticoids. Long-term follow-up indicates that AIO patients may develop ectopic ossifi cation, similar to XLH. Anti-PHEX autoantibodies were detected using both luciferase immunoprecipitation systems and fl ow cytometry, underscoring the importance of complementary methods for detecting antibodies against native conformational epitopes.

**Conclusions:**

Recognition of AIO should be particularly considered in patients with acquired FGF23-related hypophosphatemia who have undetectable PMTs, relatively mild disease activity, and concurrent autoimmune diseases.

## Introduction

Fibroblast growth factor 23 (FGF23)-related hypophosphatemic rickets/osteomalacia comprises a group of disorders caused by excessive FGF23 secretion. FGF23 acts on the renal proximal tubules through the fibroblast growth factor receptor 1 (FGFR1)/Klotho receptor complex to suppress the expression of type 2a and type 2c sodium-phosphate cotransporters, thereby inhibiting phosphate reabsorption. In addition, FGF23 decreases the expression of 1α-hydroxylase and increases that of 24-hydroxylase, leading to reduced production of active vitamin D and further lowering of serum phosphate levels [1]. Consequently, inappropriately elevated FGF23 levels cause chronic hypophosphatemia, which manifests as rickets in children before epiphyseal closure and as osteomalacia in adults after epiphyseal closure.

**Fig. 1:**
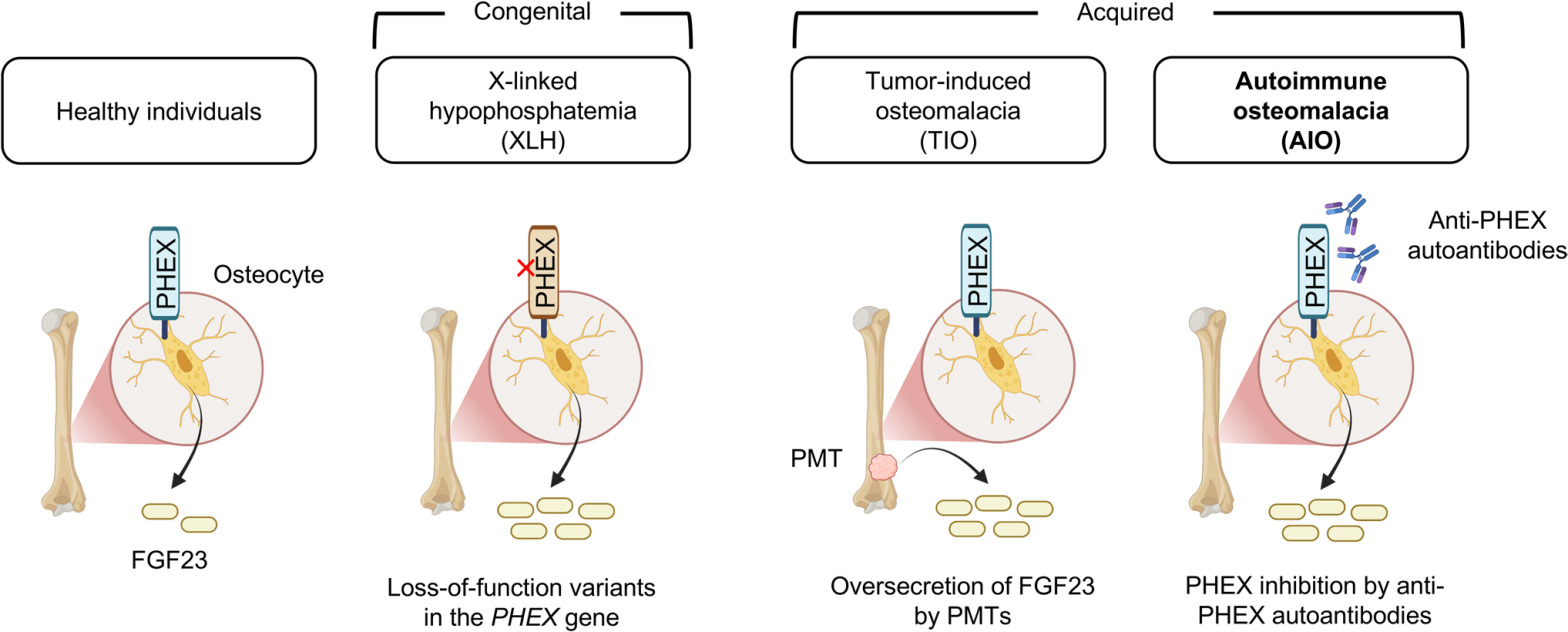
Representative disease types of FGF23-related hypophosphatemic rickets/osteomalacia. FGF23 is secreted from osteocytes, and in healthy individuals, its circulating levels are appropriately regulated by serum phosphate concentration. The most common congenital form of FGF23-related hypophosphatemic rickets/osteomalacia is XLH, which is caused by loss-of-function variants in the *PHEX* gene. The most common acquired form is TIO, which results from excessive FGF23 secretion by PMTs. In AIO, anti-PHEX autoantibodies are detectable in the serum and are presumed to inhibit PHEX protein function, thereby causing FGF23-related hypophosphatemic rickets/osteomalacia through a mechanism similar to that of XLH

The most common congenital cause of FGF23-related hypophosphatemic rickets/osteomalacia is X-linked hypophosphatemia (XLH), which is caused by loss-of-function variants in the *PHEX* gene. In contrast, the most common acquired cause is tumor-induced osteomalacia (TIO), which results from excessive FGF23 secretion by phosphaturic mesenchymal tumors (PMTs) arising in bone or soft tissue (Fig. 1).

However, even when multiple localization studies are performed in combination, PMTs remain undetectable in a substantial proportion of patients with acquired FGF23-related osteomalacia. For example, studies from Japan and abroad using fluorine-18-fluorodeoxyglucose positron emission tomography/computed tomography (FDG-PET/CT), somatostatin receptor scintigraphy with ^111^In-pentetreotide, and systemic FGF23 venous sampling have reported that PMTs are not detected in 27–40% of cases [2–4]. Although not covered by the Japanese national health insurance system, ⁶⁸Ga-DOTATATE-PET/CT offers superior sensitivity for PMT detection [5]; nevertheless, even in studies employing this modality abroad, PMTs remained unidentified in approximately 45% of cases [6].

Based on these observations, we hypothesized that an autoimmune mechanism might underlie a subset of acquired FGF23-related hypophosphatemic rickets/osteomalacia in which no PMT can be detected, and we investigated this possibility. Specifically, in addition to PHEX—the gene responsible for XLH—we screened for autoantibodies against proteins encoded by other genes known to cause congenital FGF23-related hypophosphatemic rickets/osteomalacia (*DMP1*, *ENPP1*, and *FGFR1*). Among 13 patients with acquired FGF23-related hypophosphatemic osteomalacia without detectable PMTs, we detected autoantibodies directed against the PHEX protein in five patients, suggesting that these autoantibodies contribute to disease pathogenesis [7]. This finding raises the intriguing possibility that loss of PHEX function, whether due to germline variants or acquired autoimmunity, represents a common pathway leading to FGF23 overproduction. Notably, such autoantibodies were not detected in patients with TIO or XLH, indicating that they are specific to this subgroup of acquired FGF23-related hypophosphatemic osteomalacia without detectable PMTs. We designated this novel disease entity “autoimmune osteomalacia (AIO)” (Fig. 1).

In this review, we revisit the clinical features of the five previously reported patients with AIO (Patient Nos. 9–13) [7], and provide additional insights based on newly available data.

## Clinical features of AIO and its relationship with autoimmune disease

We previously compared the biochemical parameters of the five patients with AIO to those of patients with TIO and XLH [7]. Comparison of parameters, such as serum phosphate and serum alkaline phosphatase (ALP), indicated that AIO is clinically milder than TIO and comparable in severity to XLH. These findings are consistent with the hypothesis that AIO is caused by inhibitory autoantibodies against PHEX, resulting in acquired functional PHEX deficiency.

One of the five patients with AIO had multiple concomitant autoimmune disorders, including Graves’ disease, idiopathic thrombocytopenic purpura (ITP), systemic lupus erythematosus (SLE), and antiphospholipid syndrome [7]. In addition, an ASBMR 2025 abstract originating from the United States described a patient with AIO who presented with inflammatory arthritis and autoimmune hypothyroidism [8]. These observations indicate that patients with acquired FGF23-related hypophosphatemic osteomalacia who present with relatively mild biochemical hypophosphatemia and concurrent autoimmune disease should prompt active consideration of AIO.

Several case reports have documented pediatric and juvenile patients with SLE complicated by FGF23-related hypophosphatemia, in which immunosuppressive therapy (including glucocorticoids) led to improvement not only of SLE but also of the FGF23-related hypophosphatemia [9–11]. Of the five patients with AIO, burosumab was administered to four patients (excluding one patient with impaired renal function), and improvements were observed in osteomalacia-related symptoms, serum phosphate levels, and bone alkaline phosphatase in those treated [7]. Because these reports of patients with SLE complicated by FGF23-related hypophosphatemia predate the identification of AIO, it is unknown whether those patients actually had anti-PHEX autoantibodies; nevertheless, if any of those patients did in fact represent autoimmune osteomalacia, the observed responses suggest that immunosuppressive therapy such as glucocorticoids may be effective for AIO. Unlike burosumab, which typically requires long-term (often lifelong) administration, immunosuppression has the potential to provide a more fundamental, disease-modifying treatment. However, further studies are needed to determine whether immunosuppressive therapy is effective for the treatment of AIO.

## Whole-genome sequencing in patients with AIO

WGS was performed on genomic DNA from the five previously reported patients with AIO. DNA libraries were prepared using a TruSeq DNA PCR-Free Kit (Illumina, Inc.) and sequenced as 151-bp paired-end reads on an Illumina NovaSeq 6000 platform. The resulting FASTQ files were processed with the Genomon pipeline, and reads were mapped to the UCSC human reference genome (hg38). We interrogated single-nucleotide variants/indels, splice-site variants, and structural variants in the exons of the following genes: genes associated with FGF23-related hypophosphatemic disease (*PHEX*, *FGF23*, *DMP1*, *ENPP1*, *FGFR1*, *FAM20C*, *PTH1R*, *NF1*, *HRAS*, *KRAS*, *NRAS*) and genes associated with autoimmune disease susceptibility (*AIRE*, *FOXP3*, *IL23R*, *IL12B*, *IL12A*, *TYK2*, *JAK2*, *STAT3*, *STAT4*, *IL27*, *CCR6*, *REL*, *TNFAIP3*, *NFKB1*, *TNIP1*, *ERAP1*, *ERAP2*, *IL2*, *IL21*, *IL2RA*, *IL2RB*, *IRF4*, *IRF5*, *IRF7*, *IRF8*, *CD40*, *CD28*, *CTLA4*, *ICOS*, *ICOSLG*, *PTPN2*, *PTPN22*, *UBE2L3*, *IFIH1*, *IL10*, *IL18RAP*, *FCGR2A*, *PTGER4*, *BACH2*, *CARD9*, *ZMIZ1*, *YDJC*, *TAGAP*, *PRDM1*) [12]. Variants were filtered using allele-frequency cutoffs of < 10% for homozygous variants and < 1% for heterozygous variants.

The WGS results are summarized in Table 1. All five patients with AIO harbored very rare variants in genes known to be associated with the development of autoimmune diseases that were predicted to be pathogenic. Although the allele frequency of the homozygous *ERAP2* variant is low in gnomAD v4.1.0 (0.01131), it is higher in the ToMMo 61KJPN reference panel (allele frequency 0.137304), corresponding to an approximate homozygote frequency of 1.9% in that population. Given this relatively high frequency in the Japanese reference dataset, the *ERAP2* variant is unlikely to represent a disease susceptibility valiant; nevertheless, because it was predicted to be “probably damaging” by PolyPhen-2 (v2.2.3) [13] and “deleterious” by SIFT [14], we retained it in our analysis as a potential weak disease susceptibility valiant. Notably, the patient with multiple concurrent autoimmune disorders (Table 1, Patient No. 9) carried a variant in *AIRE*—a gene implicated in autoimmune polyendocrine syndrome type 1 and central immune tolerance—raising the possibility that this variant contributed to the production of anti-PHEX autoantibodies in that patient [15–17]. Overall, these WGS findings suggest that genetic predisposition may contribute to the pathogenesis of AIO.

**Table 1:** Results of WGS in patients with patients with AIO

	Concomitant autoimmune disease	Variant gene	Genotype	Allele frequency (GnomAD v4.1.0/ToMMo 61KJPN)	Gene function and clinical relevance
No. 9	Graves’ disease, ITP, SLE, antiphospholipid syndrome	*AIRE* c.C1099T (p.P367S)	Heterozygous	0.000003722 0.000255	Plays a key role in central immune tolerance by promoting ectopic expression of tissue-restricted antigens; loss-of-function variants cause autoimmune polyendocrine syndrome type 1
No. 10	None	*IL12B* c.560T > C (p.Y187C)	Heterozygous	0.000002478 0.000131	Encodes the p40 subunit shared by interleukin-12 (IL-12) and interleukin-23 (IL-23). Genetic variants and altered expression modulate IL-12/IL-23 signaling and are associated with susceptibility to psoriasis, Crohn’s disease, and other inflammatory/autoimmune conditions
No. 11	None	*ERAP2* c.C641T (p.P214L)	Homozygous	0.01131 0.137304	An endoplasmic reticulum aminopeptidase that trims peptides for MHC class I presentation; genetic variants modulate antigen processing and have been associated with risks for several autoimmune diseases
No. 12	None	*TNFAIP3* c.2274dupC (p.K759fs)	Heterozygous	0.000001251 No report	Encodes A20, which negatively regulates NF-κB signaling and limits inflammation; polymorphisms and loss-of-function variants are associated with SLE and other autoimmune diseases
No. 13	None	*PTPN22* c.2275 + 1C > G splice donor variant	Heterozygous	0.000008774 0.000334	Encodes a lymphoid protein tyrosine phosphatase involved in signaling thresholds of T and B cells; the R620W (and other) risk variants are linked to type 1 diabetes, SLE, autoimmune thyroid disease, rheumatoid arthritis, etc.

Patient Nos. 9–13 correspond to the patient identifiers used in the previously published study [7]

The WGS results presented above reveal rare, potentially deleterious variants in genes implicated in immune tolerance and regulation across different patients. Although each identified variant alone may be insufficient to cause overt autoimmunity, the presence of such a variant in each patient suggests that it may contribute, to varying degrees, to a genetic predisposition that lowers the threshold for loss of tolerance to PHEX.

Mechanistically, the identified variants provide plausible mechanisms underlying the breakdown of immune tolerance to PHEX. AIRE promotes thymic expression of tissue-restricted antigens and is essential for negative selection of self-reactive T cells; therefore, impaired AIRE function can directly account for loss of tolerance to a specific self-antigen such as PHEX [18]. *IL12B* encodes the shared p40 subunit of IL-12 and IL-23 and regulates Th1/Th17 polarization; alterations in this pathway can enhance antigen presentation and germinal-center reactions, thereby promoting T/B helper interactions that favor class switching and affinity maturation [19]. ERAP2 is an endoplasmic reticulum aminopeptidase that trims peptides for HLA class I presentation; functional changes in ERAP2 may alter the repertoire of self-peptides presented by HLA molecules, which could increase the likelihood that otherwise infrequently presented PHEX-derived peptides are displayed and recognized by autoreactive T cells [20]. *TNFAIP3* (A20) encodes a ubiquitin-editing enzyme that provides negative feedback to NF-κB signaling; loss or reduction of A20 function lowers the activation threshold for immune responses and can enhance germinal-center formation and autoantibody production, as demonstrated in both animal models and human genetic studies [21]. *PTPN22* (Lyp) is a negative regulator of T-cell receptor signaling; while well-described functional alleles are known to modify activation thresholds and B-cell tolerance, the specific *PTPN22* variant detected in Patient No. 13 is distinct from the commonly reported canonical risk alleles [22]. Nonetheless, variation at this locus can plausibly promote survival and activation of autoreactive clones and thereby contribute to the emergence or persistence of anti-PHEX autoimmunity. In summary, our WGS data identify rare variants in genes associated with autoimmune disease susceptibility in different patients with AIO. Each patient carries a distinct predisposing variant that may lower the threshold for loss of tolerance to PHEX and, together with secondary triggers, permit the development of pathogenic anti-PHEX humoral responses.

## AIO and ectopic ossification

**Fig. 2:**
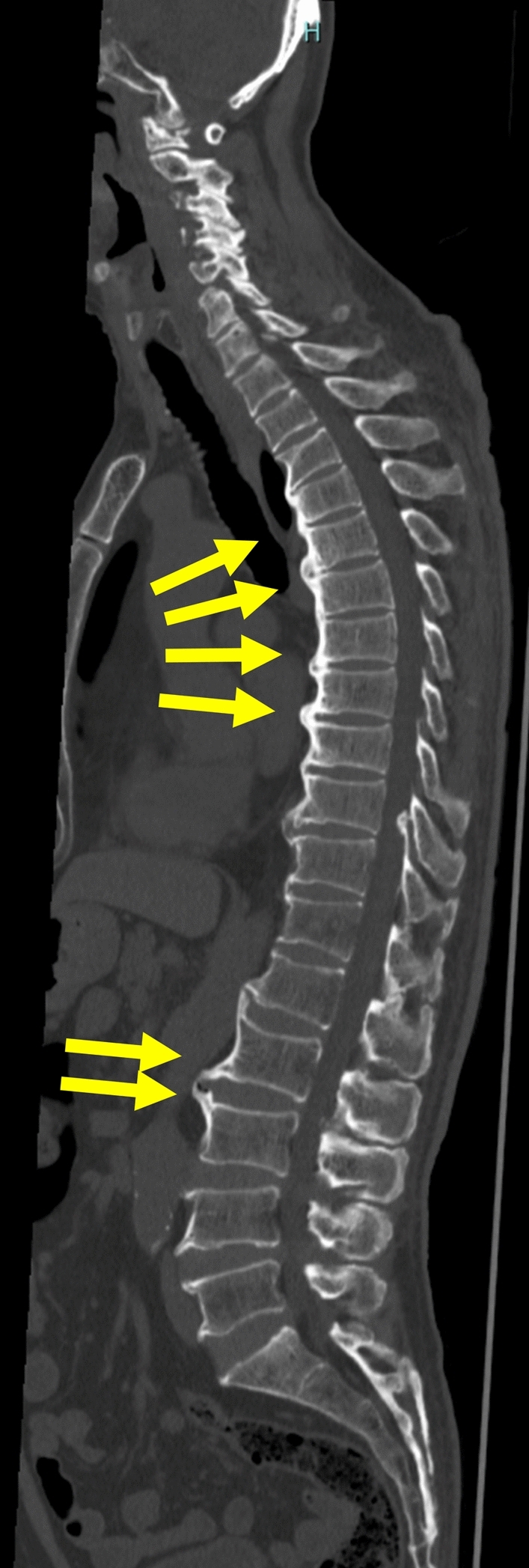
Ossification of the spinal ligaments in a patient with AIO. CT scan of the spine performed 39 years after disease onset in a patient with AIO shows ossification of the anterior longitudinal ligaments (arrows)

Thirty-nine years after disease onset, spinal computed tomography (CT) in a patient with AIO (previously reported as Patient No. 13 [7]) revealed concomitant severe ossification of the anterior longitudinal ligaments (Fig. 2). Although the underlying mechanism is not fully understood, patients with XLH are known to develop ectopic ossifications, including ossification of spinal ligaments, enthesopathies of the Achilles tendons, and enthesopathies around hip joints, which can lead to reduced quality of life [23, 24]. The onset of ectopic ossification in XLH typically occurs in the second-to-fourth decades of life [24, 25]. Because AIO and XLH share a common pathogenic feature of reduced PHEX function, it is plausible that AIO may also lead to similar ectopic ossification after decades of disease. Therefore, during follow-up of patients with AIO, clinicians should remain alert to the possibility of ectopic ossification and, if the related symptoms develop, promptly obtain appropriate imaging studies, and initiate timely therapeutic interventions (e.g., orthopedic or neurosurgical assessment and medical or surgical treatment). Be aware that treatments aimed at improving or normalizing serum phosphate levels are not expected to ameliorate these ectopic ossifications in patients with XLH and AIO, as this phenotype is observed only in certain congenital forms of FGF23-related hypophosphatemic rickets, such as XLH and autosomal recessive hypophosphatemic rickets (ARHR), suggesting that it is not a consequence of chronic hypophosphatemia or elevated serum FGF23 levels. Indeed, to date, no clinical evidence has demonstrated a preventive or therapeutic effect of conventional therapy with phosphate plus active vitamin D or of burosumab on ectopic ossification in XLH [26, 27]. In contrast, immunosuppressive therapies, such as glucocorticoids, may suppress the production of anti-PHEX autoantibodies and could therefore potentially prevent the onset or progression of ectopic ossification by targeting its underlying pathogenic mechanism in AIO. Further accumulation of patients is needed to better understand the relationship between AIO and ectopic ossification.

## LIPS assay and flow cytometry for detection of anti-PHEX autoantibodies

We detected anti-PHEX autoantibodies in patients with AIO using two complementary methods: the luciferase immunoprecipitation systems (LIPS) assay and flow cytometry. The detailed procedures for autoantibody detection by LIPS and flow cytometry have been described previously [7]. Among the five patients, four tested positive by both LIPS and flow cytometry, whereas one patient was positive only by flow cytometry [7].

**Fig. 3:**
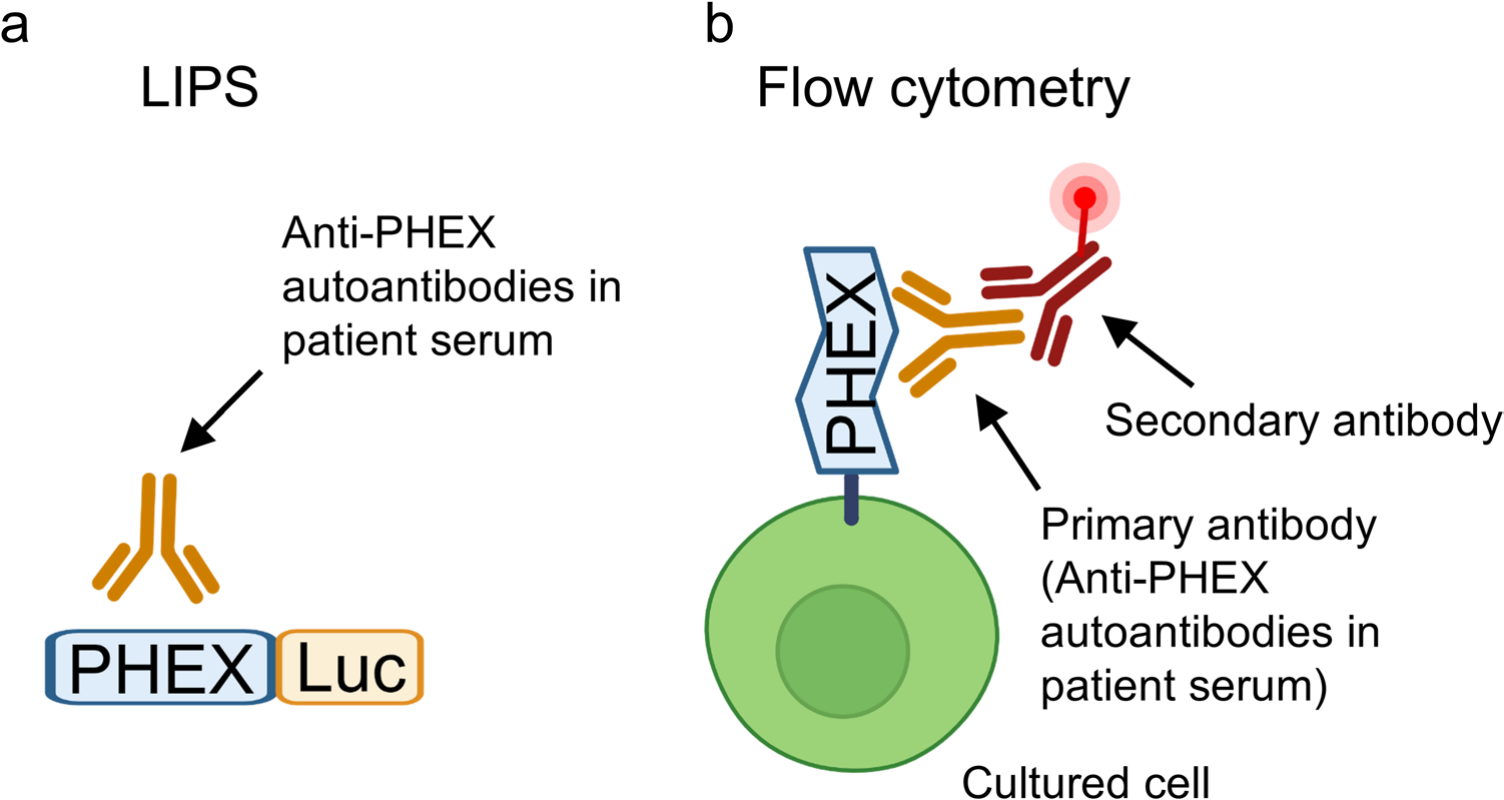
Differences in antigen conformation between LIPS and flow cytometry. **a** LIPS entails the generation of a fusion protein composed of the predicted target antigen and luciferase (Luc), with the antigen present within cell lysates. Therefore, for membrane proteins such as PHEX, the native conformation may not be preserved. **b** In flow cytometry, the antigen is presented on the cell surface in its native membrane context; thus, this method enables the detection of autoantibodies that recognize conformational epitopes on native membrane proteins

The LIPS assay entails generation of a fusion protein composed of the predicted target antigen and luciferase, allowing antibodies against intracellular, membrane-bound, and secreted proteins to be detected using the same platform. LIPS is also amenable to high-throughput screening of large numbers of samples. A limitation of the assay is that it employs cell lysates, which may not preserve the native conformation of membrane proteins; consequently, LIPS may fail to detect autoantibodies that recognize conformational epitopes dependent on the native three-dimensional structure of membrane proteins, such as PHEX (Fig. 3a).

For flow cytometry, patient sera were used as the primary antibodies, and phycoerythrin-conjugated anti-human IgG Fc antibody was used as the secondary antibody [7]. Because for membrane proteins such as PHEX, the antigen is presented on the cell surface in its native membrane context, flow cytometry enables detection of autoantibodies that recognize conformational epitopes on native membrane proteins (Fig. 3b). Thus, the antibody detected exclusively by flow cytometry in one patient most likely recognizes a conformational epitope specific to the membrane-bound form of PHEX.

As the discovery of AIO was achieved through the search for anti-PHEX autoantibodies, this combined LIPS/flow cytometry strategy provides a powerful approach for investigating autoimmune mechanisms in otherwise unexplained acquired diseases. In particular, when an acquired disorder phenocopies a known congenital disease, screening for autoantibodies directed against the protein product of the causal gene can be an efficient and targeted way to elucidate disease etiology.

## Conclusion

Among patients with acquired FGF23-related hypophosphatemic rickets/osteomalacia, those in whom PMTs cannot be localized, those with concomitant autoimmune disease, and those with relatively mild biochemical abnormalities should prompt consideration of AIO. Treatment options may include burosumab and, potentially, immunosuppressive therapies. Moreover, patients with AIO—like those with XLH—may be at risk for ectopic ossification and warrant appropriate monitoring. Although measurement of anti-PHEX autoantibodies is currently limited to the research setting, the possible presence of autoantibodies that recognize conformational epitopes dependent on the native three-dimensional structure of membrane proteins supports the combined use of LIPS and flow cytometry for autoantibody detection. Further accumulation of patients and additional studies are needed to clarify the epidemiology, pathogenesis, and optimal management of AIO.
